# HGVS Nomenclature 2024: improvements to community engagement, usability, and computability

**DOI:** 10.1186/s13073-024-01421-5

**Published:** 2024-12-20

**Authors:** Reece K. Hart, Ivo F. A. C. Fokkema, Marina DiStefano, Ros Hastings, Jeroen F. J. Laros, Rachel Taylor, Alex H. Wagner, Johan T. den Dunnen

**Affiliations:** 1 MyOme, Inc., San Francisco, USA; 2 Department of Human Genetics, Leiden University Medical Center, Leiden, The Netherlands; 3 Medical and Population Genetics Group, The Broad Institute of MIT and Harvard, Cambridge, USA; 4 GenQA, Oxford University Hospitals NHS Trust, Oxford, UK; 5 GenQA, NHS Lothian, Edinburgh, UK; 6 Department of Bioinformatics and Computational Services, National Institute for Public Health and the Environment, Bilthoven, The Netherlands; 7 EMQN CIC, Manchester, UK; 8 Manchester University NHS Foundation Trust, Manchester, UK; 9 Institute for Genomic Medicine, Nationwide Children’s Hospital, Columbus, USA; 10 Department of Pediatrics, the Ohio State University College of Medicine, Columbus, USA; 11 Department of Clinical Genetics, Leiden University Medical Center, Leiden, The Netherlands

**Keywords:** HGVS, Clinical genomics, Sequence variation, Bioinformatics, Standards

## Abstract

**Background:**

The Human Genome Variation Society (HGVS) Nomenclature is the global standard for describing and communicating variants in DNA, RNA, and protein sequences in clinical and research genomics. This manuscript details recent updates to the HGVS Nomenclature, highlighting improvements in governance, community engagement, website functionality, and underlying implementation of the standard.

**Methods:**

The HGVS Variant Nomenclature Committee (HVNC) now operates under the Human Genome Organization (HUGO), facilitating broader community feedback and collaboration with related standards organizations. The website has been redesigned using modern documentation tools and practices. The specification was updated to include guidance for transcript selection and to align with recent cross-consortia recommendations for the representation of gene fusions. A formal computational grammar was introduced to improve the precision and consistency of variant descriptions.

**Results:**

Major improvements in HGVS Nomenclature v. 21.1 include a redesigned website with enhanced navigation, search functionality, and mobile responsiveness; a new versioning policy aligned with software management practices; formal mechanisms for community feedback and change proposals; and adoption of Extended Backus-Naur Form (EBNF) for defining syntax. The specification now recommends MANE Select transcripts where appropriate and includes updated guidance for representing adjoined transcripts and gene fusions. All content is freely available under permissive licenses at hgvs-nomenclature.org.

**Conclusions:**

These advancements establish a more sustainable foundation for maintaining and evolving the HGVS Nomenclature while improving its accessibility and utility. The introduction of formal computational grammar marks a crucial step toward unambiguous variant descriptions that can be reliably processed by both humans and machines. Combined with enhanced community engagement mechanisms and improved guidance, these changes position the HGVS Nomenclature to better serve the evolving needs of clinical and research genomics while maintaining the stability that users require.

## Background

Accurate interpretations of sequence variants in clinical genomic testing depend on variants being described, communicated, and compared using conventions that are understood uniformly by researchers and clinicians. The HGVS Nomenclature [1, 2] is the de facto international standard for fostering consistency in the presentation and description of sequence variation, including clinical reports, scientific publications, variant databases, and proficiency testing/external quality assessment (PT/EQA). Although originally intended for the description of variants in humans, the HGVS Nomenclature is widely used to describe variants in other species.

Standards for representing sequence variants are crucial for ensuring consistency, accuracy, and interoperability in sequence variant data. They enable researchers, clinicians, and laboratories to communicate findings effectively, minimizing misunderstandings and errors. By adhering to established standards, sequence variants can be uniformly interpreted, compared, and integrated across various platforms and studies. This uniformity is essential for advancing research, improving diagnostic accuracy (including the efficient identification of patients eligible for therapeutic intervention), and facilitating the development of new therapies. Standards also support regulatory compliance and PT/EQA, further underscoring their critical role in the field of genetics and genomics. Additionally, in the context of software development, standards provide a clear framework for building tools and applications that can process, analyze, and share variation data seamlessly. They ensure that software developed by different groups can work together, enhancing collaboration and innovation within the scientific community.

There are several standards for representing variation within biological sequences (Table 1). While it may initially seem redundant to have multiple standards, each implements a distinct set of trade-offs made in the context of the history of a rapidly growing field. As standards evolve to support the representation of new kinds of information, they must do so in a way that preserves the interpretability of existing data and with an awareness of adjacent standards. The International System for Cytogenomic Nomenclature (ISCN) guidelines were developed in 1971 to standardize the representation of normal and abnormal cytogenomic results as inferred from karyotyping [3]; ISCN later evolved to handle new technologies such as fluorescent in-situ hybridization (FISH), chromosomal microarrays, region-specific assays, optical genome mapping (OGM) and, in collaboration with HGVS, sequencing. In 1996, the HGVS created recommendations to represent variants identified by sequencing [1, 2, 4, 5], which was still relatively low throughput and manual. Analysis requirements for next-generation sequencing (NGS) data led to the development of the Variant Call Format (VCF) in 2008 [6], which supports only genomic sequences. In 2015, the Variation Modeling Consortium (VMC) was formed to draft the Variation Representation Specification (VRS) [7], with the goal of facilitating data sharing and communication among researchers, diagnostic labs, and clinical environments through Health Level Seven (HL7) and Fast Healthcare Interoperability Resources 1 (FHIR) standards; the VMC was later expanded into the Variation Representation group within the Global Alliance for Genomics and Health (GA4GH) [8], which continues to develop the specification [9]. In 2018, the National Center for Biotechnology Information (NCBI) [10] developed the Sequence Position Deletion Insertion (SPDI) format and the Variant Overprecision Correction Algorithm (VOCA), which introduced normalization (also known as shuffling or shifting) to a fully justified representation as an alternative to right-normalized variants used by the HGVS Nomenclature or left-normalized variants used by VCF [11]. Taken together, SPDI and VOCA represent the position ambiguity of deletion-insertion variants in repeat regions more accurately than left or right normalization. VRS later adopted fully justified normalization.

**Table 1:** Comparison of sequence variant representation and presentation standards. *Coordinate systems* describes the distinct ways in which position may be defined on a reference sequence. *Normalization* describes the convention by which deletion/insertion variants are normalized (also referred to as aligned, shuffled, or shifted) in the context of sequence repeats. *Variant primitive operations* describes the distinct primitive operations that are supported by the standard; “deletion/insertion” in this column refers to the replacement of a span of sequence with another sequence, either of which might be zero-length. *Primary uses, strengths, and limitations* provides a cursory and incomplete summary of typical usage; nuances are omitted for brevity. The phrase “deletion/insertion” is used to describe deletion, insertion, and combined deletion and insertion variant types

Standard and supporting organization	Sequence types	Coordinate systems and normalization	Variant primitive operations	Primary uses, strengths, limitations
Human Genome Variation Society (HGVS) Nomenclature Human Genome Organization (HUGO)	• DNA • RNA • Protein	• Contiguous linear from sequence start or transcription start • Discontiguous exonic/intronic • Circular • Right-justified normalization	• Substitution • Deletion/insertion • Adjoined transcripts • Duplication • Extension • Frameshift • Inversion • Repeat	Predominant standard in representing variation in databases, publications, and clinical reports. Supports most sequence and variant types. as well as phased alleles and HGVS/ISCN Optionality desired by user groups necessarily creates some ambiguity
International System for Cytogenomic Nomenclature (ISCN)	• DNA	• Chromosomal bands • Contiguous linear normalization	• Aneuploidy • Derived chromosomes • Amplification • Deletion • Insertion • Duplications • Gene fusions • Inversions • Translocations • ISCN/HGVS	Long-standing standard for reporting genomic variation from karyotypes, FISH, microarray, genome mapping, and region-specific assays in constitutional and neoplasia cytogenomics. Represents variants not easily represented by other standards
Sequence Position Deletion Insertion (SPDI) National Center for Biotechnology Information (NCBI)	• DNA • RNA • Protein	• Contiguous linear • Fully justified normalization	• Deletion/insertion (as a generalization of substitutions, duplications, inversions, etc.)	Primarily used within NCBI for the precise and unambiguous representation of variants that can be represented as a combination of deletion and insertion events. The fully justified normalization using the SPDI algorithm expresses position ambiguity of variants in repeat regions better than left- or right-justified/normalized/shuffled variants
Variant Call Format (VCF) Global Alliance for Genomics and Health (GA4GH)	• DNA	• Contiguous linear • Left-justified normalization, with anchor	• Deletion/insertion • Deletion (DEL) • Breakend (BND) • Copy number (CNV) • Insertion (INS) • Inversion (INV) • Translocation (TRA)	The de facto standard file format for storing variants from NGS. Provides support for annotations, quality scores, and multiple samples within a single file. Supports only genomic sequences and file-based storage
Variation Representation Specification (VRS) Global Alliance for Genomics and Health (GA4GH)	• DNA • RNA • Protein	• Contiguous linear • Fully justified normalization	• Deletion/insertion • Adjoined sequences • Copy number • Genotype • Haplotype • Repeat	Extensible standard for representing variants on all sequence types and sharing variants between systems and in complex data structures. Primarily focused on precise representation and the minimization of ambiguity, but also supports affirmative imprecision of some concepts. Semi-structured representation limits human readability

This manuscript summarizes recent and major updates in the HGVS Nomenclature. These changes include new governance under the auspices of the HUGO, new community discussion and feedback mechanisms, and a new website that is easier to maintain and use. This release of the HGVS Nomenclature also initiates the adoption of a formal computational grammar for sequence variation and provides new guidance regarding the use of versioning to facilitate the compatibility of software. The intention of these changes is to enable the smooth and controlled evolution of the HGVS Nomenclature in order to better serve the needs of the scientific and clinical communities who rely on it.

## Methods

### Oversight and governance: incorporation into HUGO

The HGVS Variant Nomenclature Committee (HVNC) [12], a subcommittee of HUGO, maintains the HGVS Nomenclature and coordinates community feedback. The HVNC was formed from the Sequence Variant Description Working Group, a committee within the Human Variome Project (HVP), when HUGO, the HVP, and HGVS merged into one organization. “HGVS Nomenclature” was retained as the name of the standard because of its familiarity in the community and is now the preferred name for the project. Previous names — Mutnomen, VarNomen, and HGVS Recommendations — are discouraged.

The HVNC is currently composed of eleven representatives from academia, commercial, and national institutions with an interest in research and/or clinical genomics. Representatives serve 4-year terms and meet approximately six times per year. Recognizing the interconnectedness of standards efforts and the importance of consistency among them, the HVNC coordinates with the Human Genome Nomenclature Committee (HGNC), the International System for Cytogenomic Nomenclature, the Global Alliance for Genomics and Health (GA4GH) [8], and the Variant Interpretation for Cancer Consortium (VICC) [13]. Membership openings will be announced on the hgvs-announcements mailing list (discussed below) and the website.

### User communities and community engagement

The HGVS Nomenclature and the HVNC serve a diverse audience of diagnostic genetic testing laboratories, clinicians, researchers, database developers, and publishers throughout the life sciences. The HVNC seeks representation from these groups on the committee and through several communications channels.

Previously, communication with the HVNC (and its predecessor, the Sequence Variant Description Working Group) required posting questions to a Facebook group or sending an email to a dedicated email address that was accessible only to the committee’s chair. The Facebook group is now obsolete and the single-user email address has been replaced by two new email groups. The hgvs-nomenclature-announcements@googlegroups.com group is a low-volume mailing list for official announcements, such as new releases [14]. The hgvs-nomenclature@googlegroups.com is intended for community discussion; in 2024, over one hundred new members have joined and approximately three questions have been asked and answered per week [15]. Conversations for both groups are archived and publicly available without registration.

The HVNC has formalized the process by which the community may propose changes to the HGVS Nomenclature. As described on the new HGVS Nomenclature website, anyone may submit a proposal for changes to the standard. Proposals will be created in GitHub for public discussion and reviewed periodically by the HVNC. Proposals that are accepted will be drafted as changes to the HGVS Nomenclature text and refined with community input.

## Results

### Improvements to the management and presentation of the HGVS nomenclature

#### Website implementation

The previous version of the HGVS Nomenclature website was written using a mix of Markdown [16] and the HyperText Markup Language (HTML), which made it difficult to update and format consistently. Although changes were listed on a specific Versions page, the previous site presented only one version of the HGVS Nomenclature, making it difficult to investigate the evolution of the recommendations. The new site is written nearly entirely using Markdown for consistency, rendered using MkDocs [17], and automatically deployed to Read the Docs [18].

**Fig. 1:**
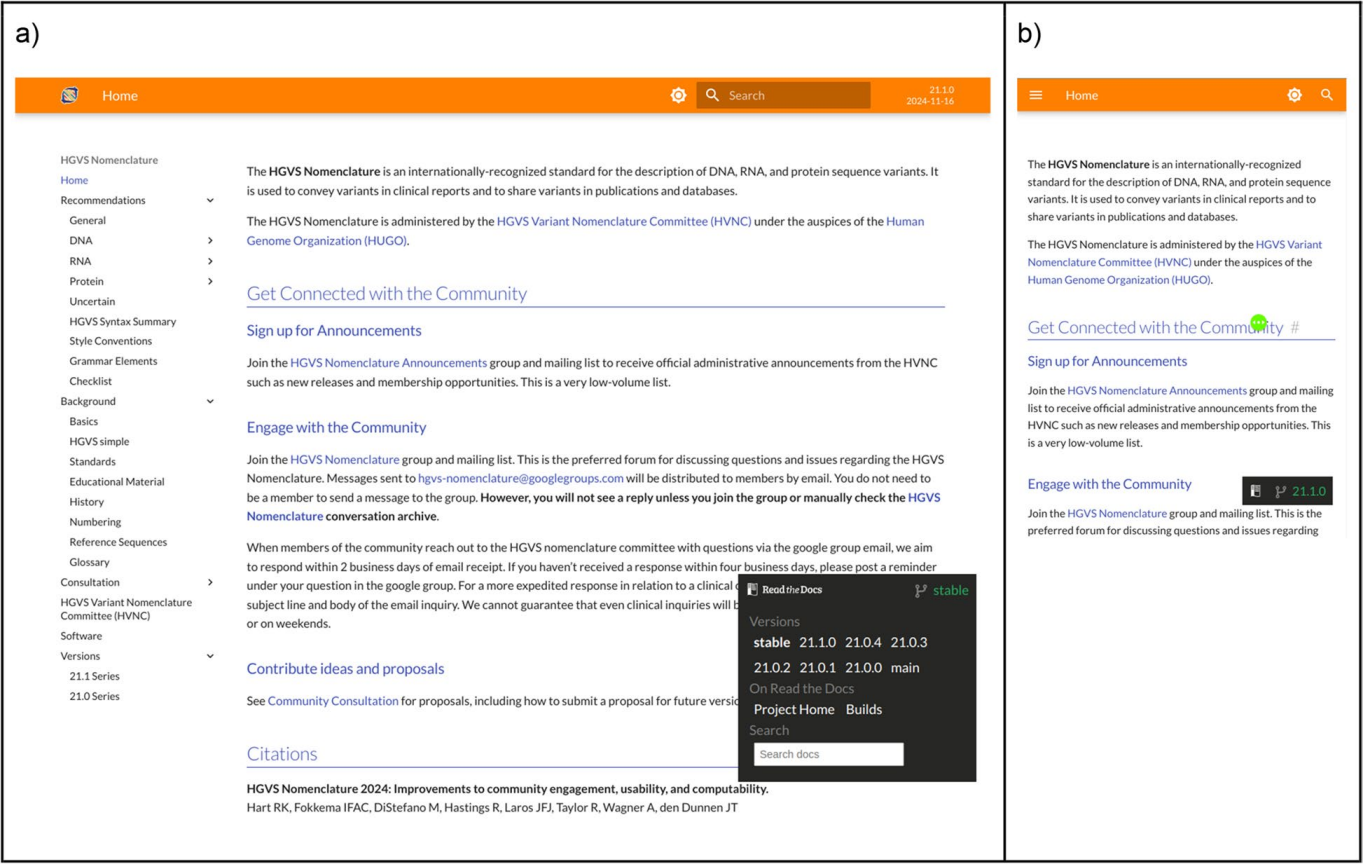
**a** The landing page for hgvs-nomenclature.org shows the “foldable” navigation menu on the left, a search bar with a clear indication of the selected version, and the Read the Docs version selector to navigate to other versions. The version selector is minimized and unobtrusive by default but expanded here to show functionality. **b** Mobile version of the landing page. The navigation menu, search features, and selected version are hidden in the “hamburger menu” denoted by the ≡ symbol

**Fig. 2:**
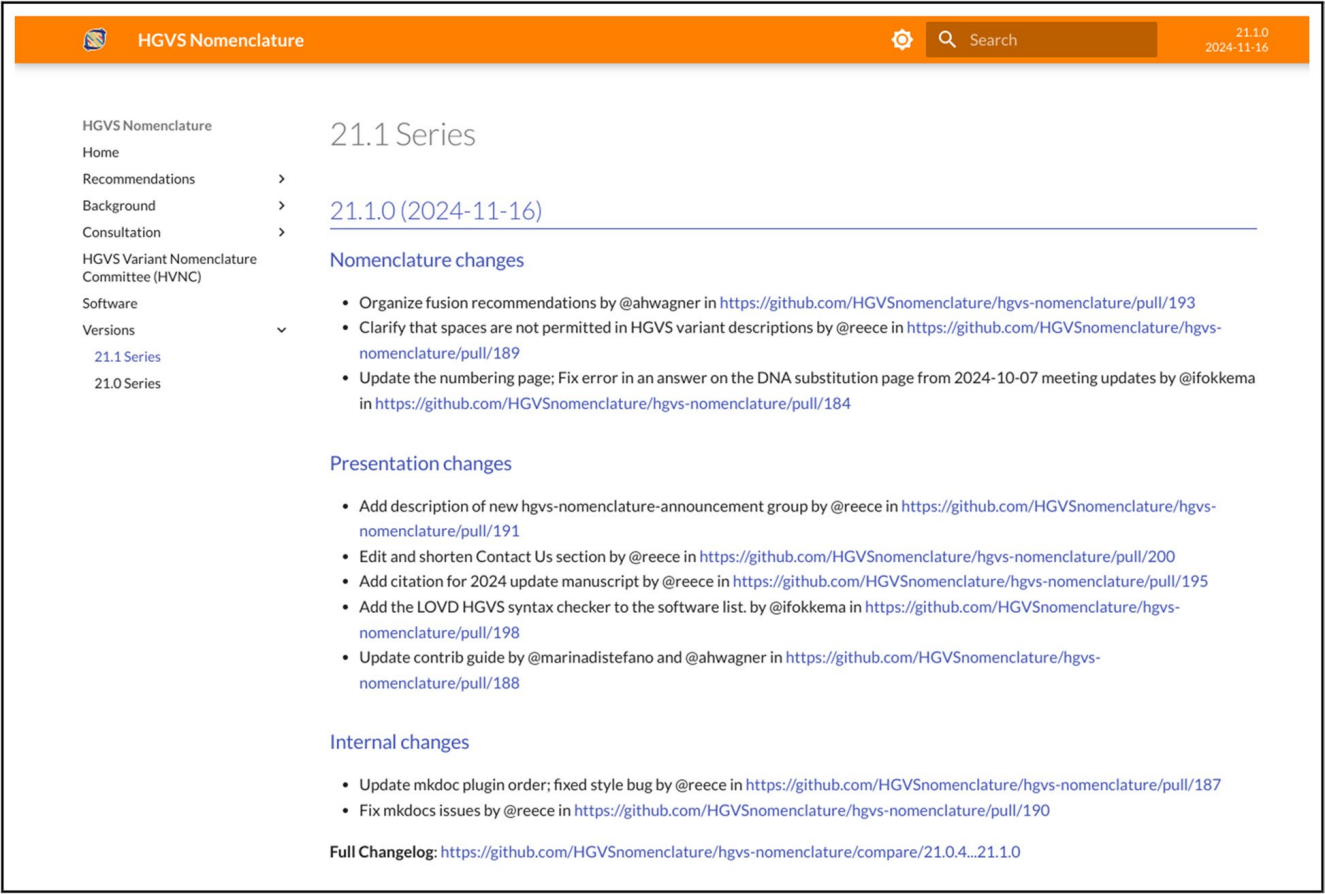
The release notes page provides details of every change to the website since the previous version

Many of the most significant new features of the HGVS Nomenclature website were created based on feedback from users. Examples include the collapsible navigation that makes it easy to understand the site layout, the version number shown in the top panel on all pages (Fig. 1), and a new versions page that itemizes changes between versions (Fig. 2). The new website enables users to easily switch between versions of the HGVS Nomenclature. Other major changes to the website include corrections of numerous small errors and inconsistencies, integrated searching with previews, the ability to navigate between different versions of the HGVS Nomenclature, and consistent styling that provides for distinguishing valid examples and invalid counterexamples (in red). The new website is responsive and remains functional across a wide range of devices, including phones, tablets, and laptops. An archived, prior version of the HGVS Nomenclature website (version 20.05) remains available [19].

#### Change processes, versioning, and release processes

The processes for updating the HGVS Nomenclature now follow conventional source code management practices. The GitHub repository [20], which stores the official documentation and code of the HVNC, is managed by a group of maintainers. Significant changes are discussed prior to implementation, and all changes are submitted as “pull requests” for review by maintainers prior to incorporation. As appropriate, the maintainers will release the aggregated changes with a new version tag and release notes. The site will be built and deployed automatically, typically within a few minutes.

The HNVC has developed a new versioning policy based on “semantic versioning” [21] that is intended to help users and adopters manage compatibility between providers and consumers of tools. Each release of the HGVS Nomenclature will be tagged with a version that has the format X.Y.Z, often called the major, minor, and patch numbers, respectively. When backward incompatible changes are made, the major version (X) will be incremented; such changes are expected to be very rare. When new features or capabilities are added, the minor version (Y) will be incremented. For trivial changes, such as typographic fixes or clarifications, the patch version (Z) will be incremented. Because the patch level carries no change in the intent of the HGVS Nomenclature, two versions may be compared by the major and minor versions alone.

The HGVS Nomenclature now recommends that *data providers* — that is, tools or databases that present HGVS variant descriptions — should advertise the version of the HGVS Nomenclature that they use. Similarly, *data consumers* — that is, websites or software tools that accept variant descriptions as input — should advertise the versions of the HGVS Nomenclature that they accept. Data consumers should use the same major version as the provider and a minor version that is greater than or equal to that of the provider; other combinations risk backward incompatibility (when the consumer’s major version is less than the provider’s major version) or lack of a feature required to parse a variant (when the consumer’s minor version is less than the provider’s minor version). Details are given on the HGVS Nomenclature Versions page [22].

#### Presentation of HGVS nomenclature grammar

Historically, the HGVS Nomenclature was presented as a set of guidelines and an informal grammar primarily aimed at human readers, providing recommendations for describing sequence variants. This approach was accessible to readers but required human interpretation and preference that is no longer suitable when building interoperable and scalable systems for the analysis of sequence variation. To enhance the precision, consistency, and interoperability of sequence variant descriptions, the HGVS Nomenclature is transitioning to the use of Extended Backus-Naur Form (EBNF) for defining its syntax [23]. EBNF is a formal notation that uses symbols and patterns to rigorously define the structure of a language, making it both transparent to human readers and precise enough for automated processing by software. This transition is essential for improving the reliability of sequence variant descriptions in computational systems, as it enables the development of tools that can automatically validate, parse, and interpret these descriptions with minimal ambiguity. By adopting EBNF, the HGVS Nomenclature is better positioned to support scalable genetic analyses, ensuring that descriptions are not only consistent across different platforms but also interoperable with other genomic standards and tools.

**Fig. 3:**
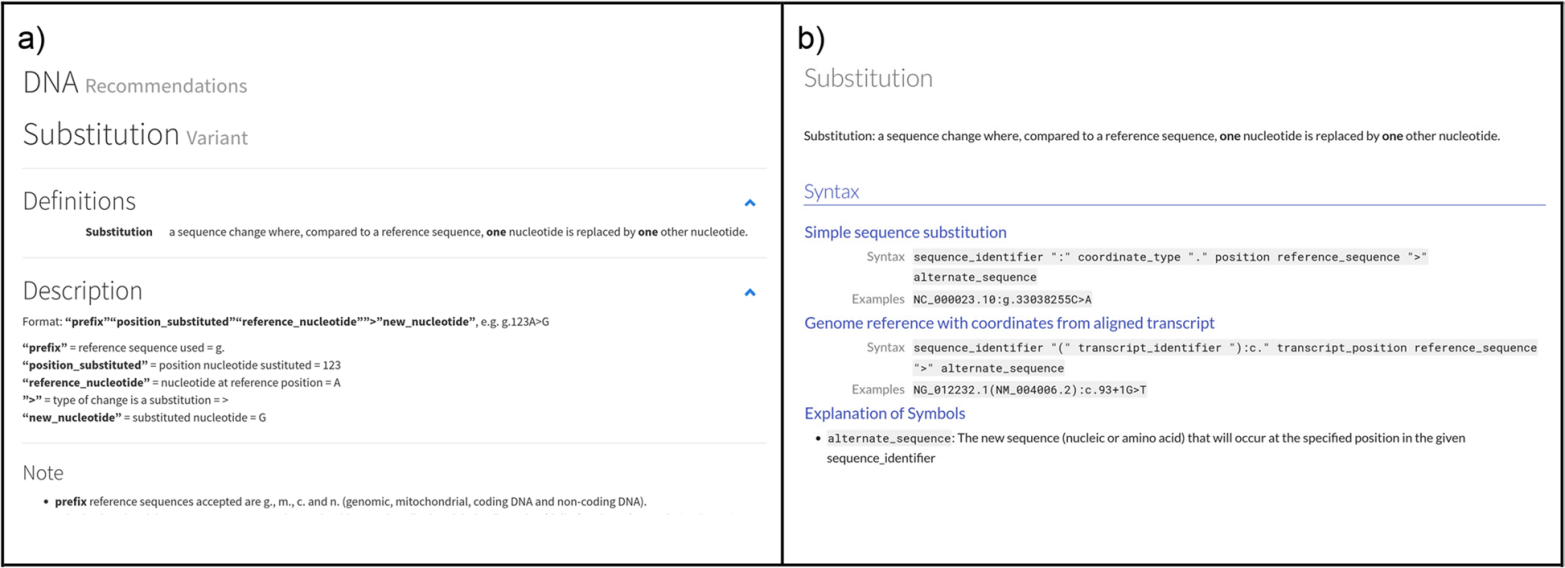
**a** Syntax for a DNA substitution from the previous website [24]. **b** Syntax for a DNA substitution from the updated website [25]. The new presentation clarifies that there are two distinct forms of a DNA substitution variant and includes an explanation of the syntactic components of each form

HGVS Nomenclature grammar is stored in a single, computer-readable file structure indexed by molecule type (DNA, RNA, protein) and variant type (substitution, deletion, etc.). All presentations of HGVS Nomenclature syntax rules are generated from this single file. Because the current HGVS Nomenclature is not easily represented as a strictly valid grammar, this release adopts the syntax of EBNF for variant descriptions without attempting to reconcile missing or ambiguous elements of the previous release. Nonetheless, the new presentation often clarifies rules that were previously difficult to interpret unambiguously. For example, the HGVS Nomenclature allows two distinct forms of DNA substitutions: a “simple” substitution on a reference sequence and a more complex form that imposes a transcript structure on an underlying genomic sequence. The existence of two forms is not obvious on the previous site but is clearly presented in the new website (Fig. 3).

**Fig. 4:**
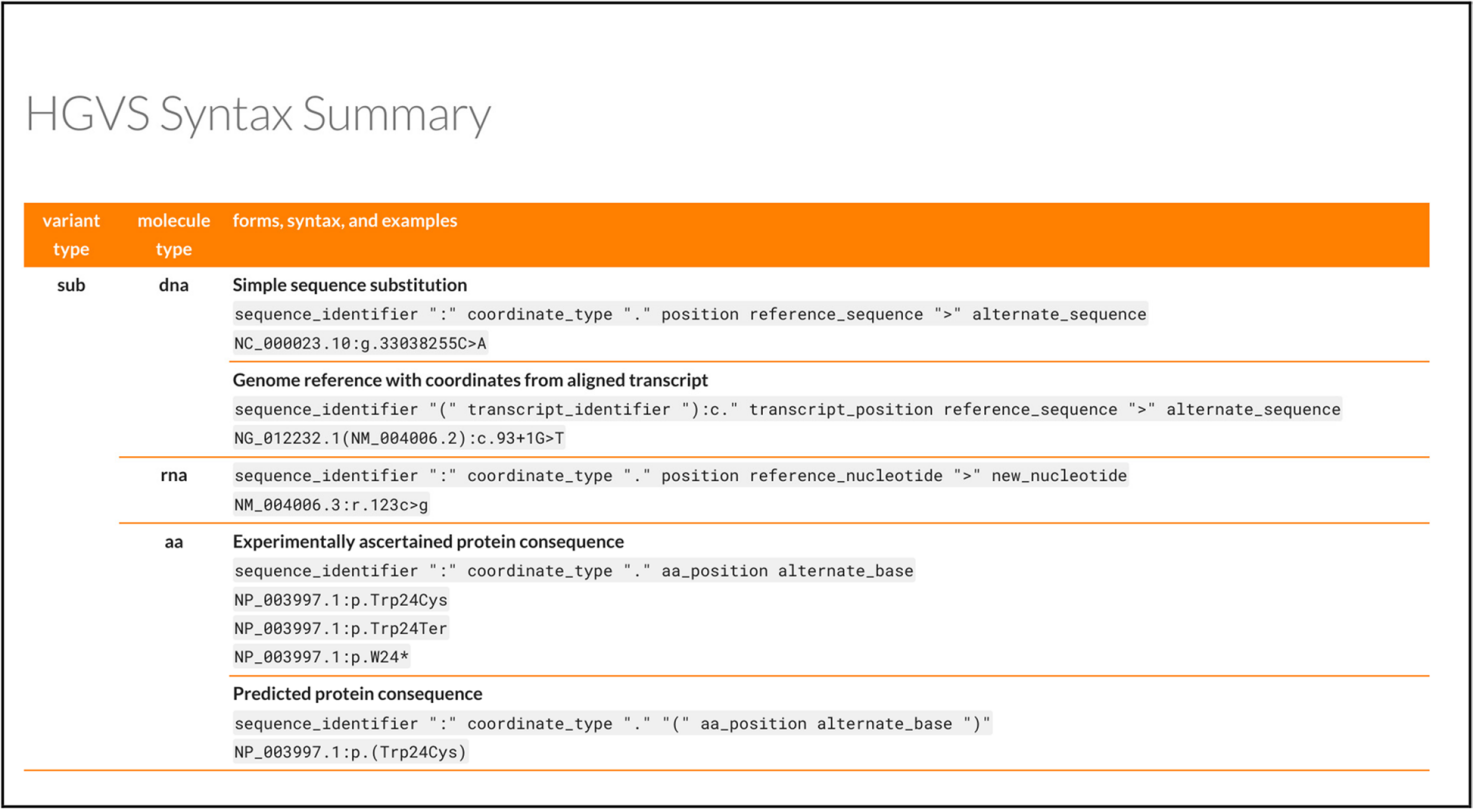
An excerpt of the new HGVS Syntax Summary pages [26]. The HGVS Syntax Summary shows all syntax rules in HGVS, organized primarily by variant type and secondarily by molecule type. This organization complements that of the website and facilitates readers to compare syntax rules. The syntax rules in the summary and molecule-specific page (Fig. 3b) are drawn from the same file, thereby guaranteeing consistency between these two views

The HGVS Nomenclature has historically been grouped by molecule type and then classified by variant type. This arrangement makes it difficult for users to identify commonalities of a variant type across molecule types. For example, it is difficult to understand how DNA, RNA, and protein forms of substitutions might appear together and users often needed to navigate at least three pages to identify the rules for a single variant type, leading to frequent requests for reorganization. Storing grammar rules in a single file enables reuse and reformatting to summarize the syntax rules by variant type, thereby facilitating comparisons across molecule types (Fig. 4).

#### List of available software

As the HGVS Nomenclature migrates towards the full adoption of a computational grammar, software will become essential to using the standard reliably and effectively. The new website includes a list of software that has been submitted by the authors and deemed to meet reasonably objective criteria [27]. Software must be a library, web API, or web user interface. Only openly available software will be listed, and well-recognized licenses by the Open Source Initiative are preferred. Currently, software may claim one or more of a set of functionality categories in order to help users find tools appropriate for their uses. Tools must also be *primarily* intended to manipulate HGVS expressions; tools that merely use HGVS expressions for input or output are not included. Tools that have tests and/or were described in a peer-reviewed publication are also preferred. To submit a tool to be included in the software list, developers can submit a pull request to the HGVS Nomenclature repository [20].

### New and updated features in the HGVS nomenclature specification

While most of the recent changes to the HGVS Nomenclature focused on how the specification is maintained and communicated, two substantive changes were made in response to timely requests from the community. A new syntax for gene fusions enables the HGVS Nomenclature to be used for adjoined transcripts. In addition, the HGVS Nomenclature 21.0 updates guidance for the selection of transcripts.

#### Aligning conventions for adjoined transcripts and gene fusions

As defined by VICC, a gene fusion is “the joining of two or more genes resulting in a chimeric transcript and/or a novel interaction between a rearranged regulatory element with the expression of a partner gene transcript” [28, 29]. An adjoined transcript results from the transcription of a gene fusion and is represented in HGVS using a double colon, e.g., NM_002354.2:r.−358_555::NM_000251.2:r.212_*279. This definition, alongside the definitions of *chimeric transcript fusions* and *regulatory fusions*, has been added to the HGVS Nomenclature glossary. Prior definitions of the terms “RNA Fusion” and “Fusion Transcript” (from community proposal SVD-WG007) have been deprecated in favor of the community-aligned term “adjoined transcript,” focusing on the precise semantics of representing two adjoined transcript sequences. A new page describing the HGVS syntax for adjoined transcripts is available under the RNA Recommendations section of the HGVS Nomenclature.

The VICC fusion nomenclature is compatible with the HGVS Nomenclature recommendations for intervening nucleic acid sequences (described as *linker sequences*) and adjoined transcripts. It also provides recommendations for an exon-based nomenclature that shares the structure of adjoined transcripts as used in the HGVS Nomenclature. In recognizing that the human genomics community has a need for representing regulatory fusions, exon-based chimeric transcript fusions, and gene-level fusions[29], the HGVS Nomenclature and ISCN now endorse the use of the VICC nomenclature for the representation of gene fusion events, in addition to their prior recommendation to use the HGNC naming conventions [30] for fusion genes.

#### Updated guidance for selection of transcripts

Historically, the HGVS Nomenclature has recommended the use of the longest transcript of a gene that represents the known biological and clinical significance of that locus. However, due to transcript sequence differences between RefSeq and Ensembl, and differences between transcripts and the primary assembly, the choice of transcript was practically challenging.

The Matched Annotation from the NCBI and EMBL-EBI Select (MANE) transcript set defines representative transcripts and corresponding proteins for human protein-coding genes [31]. MANE transcripts share identical exon structure and sequence between NCBI and Ensembl, and therefore, identifiers from those databases may be used interchangeably. Furthermore, MANE transcript sequences match the GRCh38 reference assembly identically, which avoids complications that arise with variation that overlaps alignment mismatches and gaps between transcripts and GRCh37. The MANE Select subset defines one transcript at each protein-coding locus that represents the known biology at that locus. The HGVS Nomenclature now recommends the use of MANE Select and MANE Plus Clinical transcripts where possible and appropriate. In addition, the previous recommendation to use Locus Reference Genomic sequences was withdrawn.

## Discussion

The recent updates to the HGVS Nomenclature represent a fundamental shift in how the standard is maintained and presented to the community. Taken together, these changes significantly advance the accessibility and usability of the HGVS Nomenclature.

The reorganization under the auspices of HUGO and the establishment of the HVNC play a pivotal role in maintaining and updating the nomenclature. The HVNC’s efforts to coordinate with other standards organizations, such as GA4GH, ISCN, and VICC, ensure that the HGVS Nomenclature evolves in harmony with related standards. Despite the diversity of goals and views, the individuals and organizations behind the standards continually seek to improve interoperability. The introduction of new mechanisms for community feedback, including a formal process for proposing changes, highlights the importance of community involvement in the standard’s evolution. This inclusive approach facilitates the adoption of changes that reflect the needs and insights of diverse invested users.

The redesigned HGVS Nomenclature website addresses previous usability challenges by incorporating features such as integrated search, version tracking, and a responsive design. These enhancements improve the overall user experience, making it easier for users to access, navigate, and interpret the information they need. The inclusion of a collapsible navigation menu, an intuitive search bar, and a version selector contributes to a more streamlined and accessible platform. In addition, numerous errors and inconsistencies from previous versions were resolved with this new release. These changes ensure that the website remains functional across a wide range of devices, enhancing accessibility for users worldwide.

The primary mission of the HGVS Nomenclature is to facilitate reliable communication of sequence variants, which requires that the HGVS Nomenclature is stable and consistent. Nonetheless, modifications will be required from time to time in order to address new scientific needs, resolve inconsistencies, or clarify conventions. A key goal of the HVNC is to thoughtfully balance the need for the HGVS Nomenclature to evolve while also ensuring that existing data sharing interactions are not disrupted and communicating the changes to the community clearly. The HVNC has introduced a versioning policy that balances the need for stability with the necessity of evolution. This policy allows for controlled updates to the nomenclature, ensuring that changes are communicated clearly to the community and that existing data-sharing interactions are not disrupted.

The adoption of EBNF for the HGVS Nomenclature marks a crucial advancement. This formal computational grammar provides a clear and unambiguous method for specifying the syntax of sequence variant descriptions. By ensuring that the nomenclature is both human- and machine-readable, EBNF enhances the accuracy and consistency of variant descriptions and validations. This dual readability is particularly important for enabling the development of interoperable and scalable systems that validate, manipulate, and analyze sequence variation.

Recent updates to the HGVS Nomenclature include a new syntax for gene fusion events and updated guidance for transcript selection. The new syntax for gene fusions enables the nomenclature to describe adjoined transcripts accurately, reflecting a critical need in current genomic research and clinical practice. The updated guidance for transcript selection, which now recommends the use of the MANE Select transcripts, addresses practical challenges associated with transcript sequence differences and alignment mismatches. These changes are essential for ensuring that the nomenclature remains relevant and useful in the context of advancing scientific knowledge and clinical requirements.

Transitioning from human-readable descriptions of sequence variants to a technical specification based on a formal grammar presents several challenges. One major issue is the inherent ambiguity and variability in natural language, which can lead to inconsistencies and misinterpretations when describing sequence variation. Human descriptions often rely on context and implied knowledge, which are difficult to capture in a formal grammar, leaving guidance open to inconsistent interpretation and incorrect usage. Additionally, creating a comprehensive and unambiguous formal specification requires significant effort to define and standardize all possible variant types and their representations, ensuring that they are both human- and machine-readable. This process also involves extensive community engagement and consensus-building to adopt the new specification as well as the development of tools and resources to support its implementation and use. The shift necessitates retraining and adaptation for users who are accustomed to the previous, less formalized system, which can be a substantial barrier to smooth adoption. Moreover, ensuring interoperability with existing data and systems that were designed around the old nomenclature adds another layer of complexity to this transition. The benefits of this transition, some of which are already evident in this release, outweigh the challenges associated with migrating to a formal grammar for the HGVS Nomenclature.

Professional and Compliance organizations, such as the American College of Medical Genetics (ACMG), College of American Pathologists (CAP), European Molecular Genetics Quality Network Community Interest Company (EMQN CIC), and Genomic Quality Assessment (GenQA), recommend the use of the HGVS Nomenclature (and ISCN for cytogenomic descriptions) during proficiency testing (PT) and external quality assessment (EQA) to standardize the description of sequence variants. These organizations adopt an educational approach to work globally, ensuring that genomic diagnostic testing facilities, research laboratories, and test kit manufacturers accurately and consistently communicate their findings to avoid misrepresentation of test results, which can lead to diagnostic errors and patient mismanagement. However, since some variants have multiple valid representations — for example, protein variants may use either one- or three-letter amino acid codes — exact matching of variant descriptions is often challenging. The HGVS Variant Nomenclature Committee seeks to collaborate with compliance organizations to gradually reduce optionality in the specification by providing clearer recommendations, thereby minimizing unnecessary ambiguity. Minimizing optionality will benefit the entire community, not just compliance organizations.

Conformance tests are crucial for ensuring consistent and accurate application of the HGVS Nomenclature across platforms and tools. These tests, planned as future work, will validate adherence to HGVS standards, reducing errors in sequence variant descriptions and enhancing interoperability between systems. Ultimately, these tests will uphold the integrity of the HGVS Nomenclature, ensuring it remains a trusted standard in genomics.

In conclusion, the recent updates to the HGVS Nomenclature signify a major advancement in the field of sequence variant descriptions. These changes, driven by community engagement and technological advancements, position the nomenclature as a precise, consistent, and interoperable standard that can effectively support the needs of the scientific and clinical communities.

## Conclusions

The modernization of the HGVS Nomenclature through improved governance, community engagement, and technical infrastructure represents a significant advancement for the genomics community. The transition to HUGO oversight, implementation of formal feedback mechanisms, and adoption of software development best practices have created a more sustainable and responsive foundation for maintaining this critical standard. The redesigned website, with its enhanced search capabilities, consistent styling, and clearer presentation of syntax rules, makes the nomenclature more accessible and easier to understand for both new and experienced users.

Perhaps most importantly, the introduction of a formal computational grammar marks a crucial step toward unambiguous variant descriptions that can be reliably processed by both humans and machines. Combined with the new versioning policy and guidance on transcript selection, these changes position the HGVS Nomenclature to better serve the evolving needs of clinical genomics and research communities while maintaining the stability that users require. As genomic testing becomes increasingly central to healthcare and research, these improvements to the HGVS Nomenclature will help ensure accurate and consistent communication of sequence variants across the global genomics ecosystem.

## Data Availability

Project name: HGVS Nomenclature Project home page: https://hgvs-nomenclature.org/ Source code: https://github.com/HGVSnomenclature/hgvs-nomenclature/ [20] Operating system(s): Platform independent Programming language: EBNF, Markdown, MkDocs Other requirements: Any browser License: MIT, CC0 Reference: [20]

## Abbreviations

EBI: European Bioinformatics Institute
EBNF: Extended Backus-Naur Form
EMBL: European Molecular Biology Laboratory
EMQN CIC: European Molecular Genetics Quality Network Community Interest Company
EQA: External quality assessment
FHIR: Fast Healthcare Interoperability Resources
FISH: Fluorescent in-situ hybridization
GA4GH: Global Alliance for Genomics and Health
HGVS: Human Genome Variation Society
HGNC: Human Genome Nomenclature Committee
HL7: Health Level Seven
HTML: HyperText Markup Language
HUGO: Human Genome Organization
HVNC: HGVS Variant Nomenclature Committee
HVP: Human Variome Project
ISCN: International System for Cytogenomic Nomenclature
MANE: Matched Annotation from NCBI and EMBL-EBI Select
NCBI: National Center for Biotechnology Information
NGS: Next-generation sequencing
OGM: Optical genome mapping
PT: Proficiency testing
SPDI: Sequence Position Deletion Insertion
VCF: Variant Call Format
VICC: Variant Interpretation for Cancer Consortium
VMC: Variation Modeling Consortium
VOCA: Variant Overprecision Correction Algorithm
VRS: Variation Representation Specification
